# Executive function rehabilitation and evaluation based on brain-computer interface and virtual reality: our opinion

**DOI:** 10.3389/fnins.2024.1377097

**Published:** 2024-05-14

**Authors:** Xueguang Xie, Ruihang Shi, Hao Yu, Xianglong Wan, Tiange Liu, Dingna Duan, Danyang Li, Dong Wen

**Affiliations:** ^1^School of Intelligence Science and Technology, University of Science and Technology Beijing, Beijing, China; ^2^School of Information Science and Engineering, Yanshan University, Qinhuangdao, Hebei, China

**Keywords:** executive function, rehabilitation and evaluation, brain-computer interface, virtual reality, EEG

## 1 Introduction

Executive function (EF) is an essential skill for physical and mental health, academic achievement, social development, and psychological wellbeing throughout our lives. It refers to a collection of top-down control processes used when relying on automatic responses, instinct, or intuition would be ill-advised, insufficient, or impossible (Diamond, 2013). These processes mainly include inhibitory control, working memory, cognitive flexibility, reasoning, problem-solving, and planning (Cristofori et al., 2019). However, EFs are immature from childhood through adolescence (Tervo-Clemmens et al., 2023). Additionally, various factors such as ADHD, ASD, Alzheimer's disease, and brain injury can impede EFs, leading to executive dysfunction (Negut et al., 2016). In this study, we will discuss the evaluation and training of EFs to help children and individuals with EF disorders develop these functions using advanced computer technology in the future.

Traditional EF evaluation typically involves neuropsychological tests, behavior checklists, observations, interviews, and work samples (Lezak, 2004; Cristofori et al., 2019). Recently, the evaluation method using brain-computer interfaces (BCIs), such as capturing and analyzing electroencephalography (EEG) biological signals during the performance of EF tasks, has led to more accurate results (Cipresso et al., 2012, 2013; Carelli et al., 2017). Regarding EF rehabilitation, traditional methods involve repetitive tasks using real-world materials under the guidance of healthcare professionals, which can be tedious and inconvenient. Furthermore, VR-based EF rehabilitation methods provide enjoyable and immersive virtual environments and engaging tasks, thereby enhancing the effectiveness of EF training (Liao et al., 2019). To address the issue of evaluating the effectiveness of VR-based EF training, novel methods integrating BCI-VR have been proposed (Wen et al., 2018; Duan et al., 2023). However, despite the clinical and ethical relevance of longitudinal neuropsychological evaluation in neurological disorders, there is a limited number of studies addressing the use of BCI-VR for cognitive assessment and training of patients with physical limitations. The need for expensive and complex equipment, limited and simplistic software training systems, and specific competencies to use the system and analyze data may be the main obstacles in the clinical use of BCI-VR (Carelli et al., 2017).

In this regard, we delve into the various studies using advanced computer technology for EF training and evaluation, and provide insights into current trends and strategies in various aspects.

## 2 The status of EF rehabilitation and evaluation

### 2.1 EF rehabilitation based on VR

Numerous studies have demonstrated the adaptability of virtual reality (VR) in meeting various cognitive function rehabilitation needs, particularly in the realm of executive function (EF) rehabilitation (Nir-Hadad et al., 2017; Chicchi Giglioli et al., 2021). Negut et al. (2016) discovered that VR systems offer a more engaging training experience and real-time feedback, effectively enhancing cognitive abilities during EF rehabilitation. Shema-Shiratzky et al. (2019) indicated that VR games have the potential to improve children's multitasking abilities and EFs, credited to the enjoyable stimulation provided by VR.

To cater to EF rehabilitation, researchers have designed and investigated a range of VR environments, including virtual supermarkets (Nir-Hadad et al., 2017), virtual office environments (Jansari et al., 2014), virtual kitchens (Chicchi Giglioli et al., 2019, 2021; Júlio et al., 2023), virtual cities (Jovanovski et al., 2012), virtual apartments (Tarnanas et al., 2013; Chen and Hsieh, 2018), virtual classrooms (Rizzo et al., 2006; Gilboa et al., 2015; Parsons et al., 2019), virtual games (Huang, 2020), and 360° environments (Serino et al., 2017; Borgnis et al., 2021). Participants in these studies were assigned diverse tasks such as working, cooking, shopping, and others, necessitating the utilization of EF skills such as planning, decision-making, impulse control, working memory, multitasking, and problem-solving. Notably, researchers observed significant improvements in participants' EF abilities following the VR interventions through performance measurements.

A recent review by Borgnis et al. (2022) concluded that VR-based tools hold promises as solutions for ecologically assessing and rehabilitating EFs. However, challenges remain in terms of accessibility, user-friendly operation of VR devices, and personalized rehabilitation (Elbamby et al., 2018). Additionally, Newman et al. (2022) proposed that the level of realism in VR scenes can influence users' emotions and perceptions, highlighting the potential benefits of enhancing realism for EF training.

### 2.2 EF evaluation based on BCI

Nowadays, an increasing number of human-computer interaction (HCI) methods, such as eye tracking (Katona, 2021a, 2022, 2023) and capturing brain activity signals (Katona and Kovari, 2018), are being utilized to assess various learning and training processes, and they have shown promising performance. Among the different modalities for capturing brain activity signals, EEG stands out as the most widely used modality in the field of BCI cognitive assessment research, in comparison to functional near-infrared spectroscopy (fNIRS; Chai et al., 2024), electromyography (EMG), and electrocorticography (ECoG). This is primarily attributed to EEG's advantages of high spatiotemporal resolution, low cost, portability, and real-time monitoring capabilities (Liu et al., 2017). Therefore, in this study, we focus on investigating the EF evaluation method based on EEG.

Alturki et al. (2020) proposed a method based on wavelet transform to extract features from EEG signals for diagnosing neurological disorders. Furthermore, frequency band features of EEG are commonly used to identify and classify cognitive impairments based on their association with cognition and neurological disorders (Newson and Thiagarajan, 2019). Viviani and Vallesi (2021) found that the theta band represents a potential target frequency for enhancing EFs. Perone et al. (2018) utilized a regression analysis method to examine the relationship between resting-state EEG activity and EF and discovered that differences in the theta/beta ratio were associated with EF. Zhang et al. (2019) analyzed the relationship between EEG spectral power and EF tasks in participants with ADHD and inferred the prognostic value of resting EEG, which may be biomarkers of neuropsychological functions. The findings from various studies highlight the potential value of EEG in evaluating neuropsychological functions.

Building upon signal analysis, several cognitive classification algorithms have been investigated. Mandal et al. (2020) used support vector machines (SVM) for EEG binary classification at different cognitive levels. However, SVM cannot handle this dynamic property. Therefore, Sakhavi et al. (2015) proposed an architecture utilizing convolutional neural networks for classification based on the dynamic energy representation of EEG. Furthermore, Zhu et al. (2020) designed a lightweight Convolutional Neural Network that successfully detected signs of sudden death in patients. These studies have unveiled novel research prospects for the precise assessment of EF. However, further improvement of these algorithms is necessary to meet the demands for precise EF evaluation.

### 2.3 BCI-VR application in cognitive rehabilitation and evaluation

Since there is limited research specifically focused on BCI-VR for EF rehabilitation training, we expanded our scope and explored the literature on BCI-VR in cognitive rehabilitation. They share common aspects and provide insights that can contribute to our understanding of BCI-VR applications in EF rehabilitation. Recently, Wen et al. (2018) anticipated that the integration of VR and BCI in cognitive training holds great potential to effectively intervene and improve the cognitive functions of patients, including EF evaluation and rehabilitation. Besides, the researchers overviewed the recent studies and proposed that BCI-VR systems could increase the enthusiasm of the individual in training, and provide more effective feedback, and promote recovery of brain function (Wen et al., 2021). Legrand et al. (2011) made efforts to use EEG to obtain physiological indices to measure the performance of VR training. Wan et al. (2021) measured the impacts of VR games on cognitive ability using EEG signals and performance data. Lee et al. (2022) utilized time-domain EEG analysis to evaluate the effects of a person's mental workload during VR training, demonstrating the feasibility of BCI-VR in cognitive rehabilitation. Robledo-Castro et al. (2023) reviewed cognitive training programs based on artificial systems and digital technologies on EFs and concluded that recent initiatives have started incorporating user-machine interfaces, robotics, and VR, although there is still limited research on their effects. Katona proposed that cost-effective CogInfoCom-based systems, like the fusion of HCI-based systems and 3D VR environments hold tremendous potential. However, it is important to take into account that the development, operation, and maintenance of these increasingly complex systems will pose new challenges (Katona, 2021b).

Overall, these studies demonstrated the developmental trends in EF rehabilitation based on BCI-VR technology. Nevertheless, the application of BCI-VR in EF rehabilitation faces several challenges, including enhancing technological practicality, user-friendliness, and operability, as well as addressing concerns regarding ecological validity, individual variability, and effectiveness. Further investigation is warranted to explore methodologies that can augment the efficacy of BCI-VR.

## 3 Challenges of EF rehabilitation and evaluation based on BCI-VR

As mentioned in the previous chapter, there is significant potential for EF training and evaluation utilizing BCI-VR technology. However, numerous challenges need to be addressed, including hardware device limitations, virtual environment complexities, and data analysis intricacies.

### 3.1 Complex, imprecise, and costly hardware

During EF training, participants wear all-in-one BCI-VR devices, such as the combination of the HTC VIVE VR device and the NEUSEN W-64 EEG amplifier, to perform tasks. However, the excessive weight of devices can lead to inconvenience and fatigue when worn for a long time. There is a need for further improvement in terms of their portability. Additionally, movement can introduce instability and latency in EEG signal data, compromising the accuracy of measurements (Wen et al., 2021). Moreover, operating these devices is complex and requires highly trained professionals. The use of controllers or a mouse to operate the devices diminishes the immersive advantages and results in poor operability (Robledo-Castro et al., 2023). Furthermore, the high cost of these devices limits their widespread application.

### 3.2 Limited and simplistic virtual environments

The current virtual environments for EF training primarily focus on simple scenarios like virtual kitchens or virtual shopping. These scenes are still relatively unrealistic, monotonous, and tedious, which limit performance and diminish the ecological validity of EF rehabilitation (Elbamby et al., 2018; Newman et al., 2022). Creating high-quality virtual scenes, on the other hand, demands substantial time, resources, and technical expertise. Moreover, there is a challenge in addressing personalized needs within repetitive VR scenes.

### 3.3 The lack of automation in program design and evaluation

The current dependence on manual methods for designing rehabilitation training tasks and evaluating the effectiveness of rehabilitation is characterized by time-consuming and subjective processes, leading to inefficiencies. The lack of automation impedes the customization of training programs based on individual needs and hinders the objective evaluation of progress (Carelli et al., 2017). Furthermore, the absence of automated systems limits the efficient analysis of large datasets and real-time intervention capabilities (Alturki et al., 2020). The deficiency in automation in EF program design and evaluation hampers scalability, standardization, and the optimization of cognitive rehabilitation outcomes.

## 4 Trends of EF rehabilitation and evaluation based on BCI-VR

In addressing the aforementioned issues, we believe that machine learning and artificial intelligence can play a significant role in enhancing the accuracy and usability of BCI-VR methods for EF rehabilitation and evaluation.

### 4.1 Designing a low-cost, lightweight, and user-friendly device

Developing online and remote rehabilitation, where scene rendering, data analysis, and storage are performed in the cloud, will significantly reduce equipment purchase costs for individual users. Additionally, patients can receive virtual rehabilitation therapy at home or other locations, thereby reducing time and geographical limitations (Yin et al., 2022). This mode also enables real-time monitoring and remote guidance, allowing healthcare professionals to remotely track patient progress, make necessary adjustments, and provide support. Furthermore, combining eye-tracking signals can provide more precise user behavior data and feedback, while data fusion with EEG can enhance data accuracy by leveraging multiple data sources (Cipresso et al., 2013).

### 4.2 Enhancing realism and personalization of virtual environments

Advanced VR-GS technology based on 3D Gaussian splatting (Jiang et al., 2024), including VR auto-generation, high-resolution graphics, and physical simulation, has the potential to improve the simulation of real-world environments and behaviors, thereby enhancing effectiveness and ecological validity. Moreover, with the development of multimodal large language models and artificial intelligence-generated content (AIGC; Cao et al., 2023), it has become possible to personalize virtual scenes for each patient. Specifically, customized scenes can be automatically generated based on patients' language, provided images or videos, as well as their preferences. Moreover, combining gaze-based interaction technology based on eye-tracking can optimize interface design and rendering (Chen et al., 2023), which in turn reduces user cognitive load and enhances the user experience.

### 4.3 Achieving data-driven program design and evaluation

Developing data-driven adaptive EF training program design and evaluation based on BCI-VR will bring about more accurate training programs with less human intervention. In addition, taking into account personal preferences and individual differences, such as age, gender, etc., tailoring personalized and adjustable training programs will become a research trend. Finally, further development of a real-time automated system for multimodal fusion data analysis and feedback algorithms will make it possible to achieve immediate effect feedback and further action guidance, which will accelerate EF rehabilitation.

## 5 Conclusions

In summary, this study explores the current state of research on EF rehabilitation and assessment and highlights the great potential of BCI-VR technology in effectively improving individuals' EF. Furthermore, we analyze the challenges and future directions for BCI-VR systems. Although BCI-VR technology in EF training currently faces limitations in terms of hardware, visual environment, and data analysis, we believe that with technological advancements, such as online systems, VR-GS, AIGC, and multimodal fusion analysis algorithms, these issues will be alleviated and resolved. In the future, more convenient, effective, diversified, and economical systems will provide greater assistance to a larger number of patients with executive dysfunction.

## Author contributions

XX: Investigation, Writing – original draft. RS: Investigation, Writing – review & editing. HY: Writing – review & editing. XW: Writing – review & editing. TL: Writing – review & editing. DD: Writing – review & editing. DL: Writing – review & editing. DW: Writing – review & editing, Funding acquisition, Supervision.
